# Three-Dimensional Face Reconstruction Using Multi-View-Based Bilinear Model

**DOI:** 10.3390/s19030459

**Published:** 2019-01-23

**Authors:** Liang Tian, Jing Liu, Wei Guo

**Affiliations:** The Key Laboratory of Augmented Reality, College of Mathematics and Information Science, Hebei Normal University, No.20 Road East, 2nd Ring South, Yuhua District, Shijiazhuang 050024, Hebei, China; mrtianliang@126.com (L.T.); eric_lj@126.com (W.G.)

**Keywords:** 3D reconstruction, 3D vision, multi-view-based bilinear model, model matching, 3D shape modeling

## Abstract

Face reconstruction is a popular topic in 3D vision system. However, traditional methods often depend on monocular cues, which contain few feature pixels and only use their location information while ignoring a lot of textural information. Furthermore, they are affected by the accuracy of the feature extraction method and occlusion. Here, we propose a novel facial reconstruction framework that accurately extracts the 3D shapes and poses of faces from images captured at multi-views. It extends the traditional method using the monocular bilinear model to the multi-view-based bilinear model by incorporating the feature prior constraint and the texture constraint, which are learned from multi-view images. The feature prior constraint is used as a shape prior to allowing us to estimate accurate 3D facial contours. Furthermore, the texture constraint extracts a high-precision 3D facial shape where traditional methods fail because of their limited number of feature points or the mostly texture-less and texture-repetitive nature of the input images. Meanwhile, it fully explores the implied 3D information of the multi-view images, which also enhances the robustness of the results. Additionally, the proposed method uses only two or more uncalibrated images with an arbitrary baseline, estimating calibration and shape simultaneously. A comparison with the state-of-the-art monocular bilinear model-based method shows that the proposed method has a significantly higher level of accuracy.

## 1. Introduction

Three-dimensional (3D) face reconstruction is one of the most fundamental and challenging problems in 3D vision systems, as it plays an important role in many fields such as face geometry measurement [1,2,3], face recognition [4,5,6], face component transfer [7,8] and face replacement [9,10]. In the recent decades, significant progress has been made in 3D face reconstruction and a number of methods have been proposed in the literature. These approaches can be divided into two categories: active and passive. On one hand, the active method indirectly obtains high-quality 3D facial model using depth maps captured from sensors, such as a laser scanner device ($Cyberware^{TM}$) or depth camera (Microsoft$Kinect^{TM}$) [11]. Unfortunately, the laser scanner device is harmful to human eyes and is too expensive for many applications. Moreover, sensor errors and the properties of the face surface mean that depth maps from depth sensors are often noisy [12]. Additionally, the active method also needs to take sufficient time to complete the scanning process, during which the face must be kept still for a few seconds. Hence, it is more suitable for static application scenes.

On the other hand, the passive method can obtain facial shapes using image sequences [13] or 3D morphable models [7]. It is generally used in dynamic application scenes. However, the image sequence-based passive methods heavily rely on how the scene is presented and contain error matches caused by luminance variation. They also fail in texture-less and texture-repetitive regions where there is not enough visual information to obtain the correspondence. Moreover, they cannot deal satisfactorily with fast motion and a wide range of distances. On the contrary, 3D morphable model-based methods do not suffer from ambiguities in texture-less and texture-repetitive regions. However, the number of feature points is very limited, and the methods use the 2D image location information of face feature points to obtain the identity and expression weights, which often leads to low quality and noise susceptibility.

It is clear that each 3D face reconstruction method is limited in some respects where other approaches may be effective. Therefore, fusing different methods using their complementary characteristics would undoubtedly make the obtained 3D face reconstruction more robust and improve the quality. Here, we extend the traditional monocular bilinear model to the multi-view-based bilinear model by fusing the complementary characteristics of the shape from stereo matching and the 3D morphable model to obtain an accurate and robust 3D face mesh. The feature prior constraint and texture constraint are formulated into our framework to obtain a much higher precision estimation than that demonstrated in previous papers. The main contributions are as follows:

1. Feature pixels extracted from multi-viewpoints are used as the feature prior constraint, which is treated as a face shape prior to estimating an accurate 3D face mesh contour. The rigid transformation of the face mesh as well as the identity and expression weights in the multi-view-based bilinear model can be estimated by minimizing the matching error between the extracted feature pixels and the projections of the landmarks in multi-viewpoints. The proposed constraint can estimate accurate contours of the generated 3D face mesh even with the effect of occlusion, where previous methods often failed. This is possible because when some parts of the face mesh are invisible in one viewpoint, they may be visible in other viewpoints, which can provide accurate feature pixels to restore the facial contours.
2. An adaptive texture constraint is incorporated to extract a high-precision 3D facial shape, where traditional methods fail because of the limited number of feature pixels or the mostly texture-less and texture-repetitive input images. Meanwhile, it fully explores the implied 3D information between multi-view images, which also enhances the robustness of the results. The adaptive texture constraint is defined as the weighted sum of squared texture differences between corresponding stereo-match pixels in the multi-viewpoint images. It assumes that the stereo-match pixels in the projected face region between multi-view images should be projected from the same vertex in the 3D face mesh and have a similar texture distribution.

The accuracy of the method is evaluated on ground-truth data and compared against the state-of-the-art 3D face reconstruction method. It is shown how the feature prior and texture constraints from the multi-view images facilitate the extraction of a 3D face mesh. Additional experiments validate the pose invariance of the method on real-world face images. The proposed method works directly on uncalibrated multi-viewpoints with an arbitrary baseline, to estimate the calibration and 3D face mesh simultaneously by a GPU-based optimization.

The remainder of this paper is organized as follows: Section 2 gives a summary of various methods used for 3D face reconstruction. The details of the proposed method are discussed in Section 3, while the evaluation results are presented in Section 4. Section 5 gives some conclusions for the future work. Note that for notation clarity, in this paper, we focus only on restoring the 3D face mesh from three viewpoints. However, the proposed method can easily be used to handle 3D facial reconstruction from any multi-views.

## 2. Previous Work

In recent years, the image sequence-based passive 3D face reconstruction method is regarded as a hot area of 3D vision system [13]. According to the type of image information, it recovers the 3D facial mesh from shading, motion, stereo matching, and the 3D morphable mode.

The shape from motion-based method (SFM) uses the image sequence to estimate the 3D structure of the human face in a controlled motion [14]. It estimates the camera calibration parameters using the matching feature pixels in each frame image and then restores the depth information of the human 3D face geometry. Moons et al. [15] used a universal model to reconstruct a 3D face model from a video sequence. Torresani et al. [16] estimated the 3D model of non-rigid objects from a video sequence recording human speech and used the probabilistic principal components analysis method to learn the 3D model of human face. Garrido et al. [17] proposed a novel algorithm for the automatic creation of a personalized high-quality 3D face rig of an actor from just monocular video data. Their rig is based on three distinct layers that allow them to model the actor’s facial shape as well as capture his person-specific expression characteristics at high fidelity. However, because the method relies heavily on the local texture information to find matching feature pixels between frames, SFM does not perform well in dynamic scenes and is easily affected by illumination variation, fast movement, motion blurring and occlusion between adjacent frames.

The shape from shading-based method (SFS) estimates the 3D facial face shape through a nonlinear partial differential equation, which indicates the relationship between the intensity of each 2D image pixel and normal reflectivity. Tanasai et al. [18] recovered a 3D face model from a single shaded face image. The key to their work was to find a possible relationship between an image subspace and a 3D subspace. Kemelmacher et al. [19] combined the shading information along with prior knowledge (such as the lighting conditions, the reflectance characteristics of the object, and boundary conditions) of a reference model to obtain the 3D shape of a novel face from a single image. This prior information is part of the sought 3D shape and is often not available. This limits the applicability of the SFS method to restricted setups.

The goal of shape from stereo matching-based method (SFSM) is to estimate a high-resolution dense depth map by finding corresponding pixels in image pairs taken simultaneously from different viewpoints [20]. Sun et al. [21] proposed a fast framework for 3D face reconstruction by using uncalibrated photometric stereo. With a reference face model, lighting parameters were estimated from input face images lit by unknown illumination, which can be used in classical photometric stereo to estimate the surface normal and albedo. Nigam et al. [22] proposed an automatic 3D face reconstruction approach from rectified stereo images. Three enhancements adaptive histogram equalization, horizontal gradient ordinal relationship pattern and steerable filter were employed to improve the contrast of face images, handle poor illumination and reduce noise. The SFSM methods heavily rely on the features (e.g., color, color gradients, edges), the radiometric variations and the presentation of the human face. However, the human face is relatively texture-less or texture-repetitive except for a few edges (such as wrinkles, the mouth, and the eyes). Where there is not enough visual information to obtain a correspondence, the extraction of high-quality surfaces with general correlation-based methods is virtually impossible. Hence, the results of the SFSM method are relatively low in terms of accuracy and contain error matches caused by luminance variation and occlusion [23].

Another passive method indirectly obtains facial reconstruction using a 3D morphable model (3DMM) that does not suffer from ambiguities in texture-less and texture-repetitive regions. As a classic statistical model of 3D facial shape, 3DMM derives a morphable face model, learned from a database of facial scans, as a vector space representation that is used to describe the procedure of constructing a 3D face. The 3DMM has two significant benefits: on one hand, a point-to-point correspondence between the reconstruction and all other models; on the other hand, modeling underlying transformations between face types (amazement, fear, smiling, etc.). Blanz et al. [24] derived a 3D morphable model based on a set of 200 3D face models describing shapes and textures. Their method computed the correspondences using optical flow between the reconstructed face and the input still image. Although reconstructing realistic 3D face reconstruction from a single still image, it is highly computationally expensive and only effective under constrained settings because of the non-linear optimization. To overcome this weakness, Vlasic et al. [25] used a multilinear model to represent the combined effect of identity and expression variation on the facial shape. Dale et al. [26] proposed a 3D multilinear model to track the corresponding 3D facial geometry in both space and time from the source to the target face performance in a video sequence. Bolkart et al. [27] showed how such a multilinear model can be estimated directly from 3D scans using joint optimization over the model parameters and groupwise registration of 3D scans.

Among the plethora of 3DMM methods [28], the monocular bilinear model [7] is probably the most well-known and widely applied method used to reconstruct 3D face shapes. It assembles the dataset into a rank-three data tensor, which is arranged in an obvious fashion so that it represents the 3D face by forming linear combinations of the prototypes. Moeini et al. [29] obtained expression insensitive face recognition from only a 2D single image in a gallery including any facial expressions. However, because it represents the 3D face mesh from a single image, the required information for high-precision 3D face reconstruction used in the monocular bilinear model (such as shape, texture, illumination conditions and camera attributes) is limited, so the recovery of 3D face geometry is ill-posed and uncertain. Furthermore, the feature point detection in the monocular bilinear model is a crucial task and may be inaccurate, especially when the image contains various lighting conditions, facial expressions, wrinkles and so on. Moreover, the feature pixels are affected by the occlusion, which results in low accuracy and affects the shape estimate [30]. So, obtaining a 3D face reconstruction with a single face image is still an ill-posed problem.

Instead, multi-view geometry is a common procedure to reconstruct 3D shapes from several single images or video frames. It has become a hotspot in recent years. The 3DMM based on multi-view can significantly utilize redundant data from multiple images of a person to obtain robustness and reliability. Zhu et al. [31] proposed a robust method using multi-feature framework which included SIFT feature, pixel intensity and contours. Because the SIFT feature is invariant to uniform scaling, orientation, and partially invariant to affine distortion and illumination changes, their method can locate the facial components successfully. Lin et al. [32] proposed an improved 3DMM for multi-view 3D face reconstruction. They significantly reduced tens of thousands of image intensities in the 3DMM to dozens of sparse facial feature points. However, due to the small number of feature points, the reconstruction process is prone to overfitting. Booth et al. [33] improved the texture of 3DMM by learning in-the-wild feature-based texture model. Meanwhile, Tran et al. [34,35] proposed an innovative framework to regress robust and discriminative 3DMM representation from a large set of unconstrained multi-view face images. Fyffe et al. [36] presented a multi-view reconstruction method that directly obtains a complete high-fidelity head model with a consistent facial mesh topology. While existing techniques separate shape estimation from facial tracking, their framework jointly optimizes 3D constraints and provides consistent mesh parameterization. Dai et al. [37] propose a novel “coarse-to-fine” multi-view 3D face reconstruction method by taking advantage of the complementarity between facial feature points and occluding contours. Multi-view face images with visual angle differences were employed to calculate the 3D coordinates of facial feature points to generate 3D face models. However, these methods generally depended on a generic 3D face model, while a lot of the texture information is always ignored.

According to the above discussion, each 3D face reconstruction method is limited in some aspects, and other methods may be effective. Therefore, the fusion of different methods and the use of their complementary features will undoubtedly make the 3D face reconstruction more robust and improve the quality.

## 3. Methods

**Figure 1:**
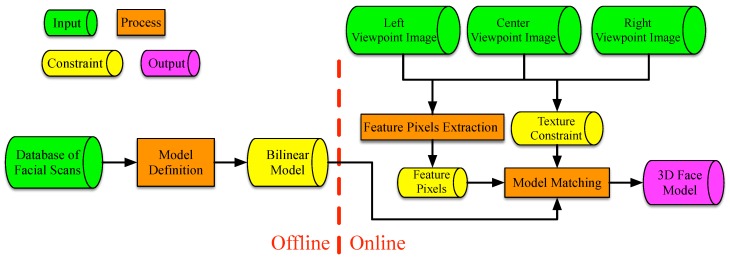
The conceptual flow diagram of the proposed method.

The proposed method can be partitioned into three phases: model definition, feature pixels extraction and model matching. The inputs are the texture images ($I_{L}$, $I_{C}$ and $I_{R}$) of the left, center and right viewpoints, and the output is the generated 3D face reconstruction mesh. As shown in Figure 1, during the model definition, the FaceWarehouse [7], a database of 3D face meshes for visual computing, is used to build the face bilinear model. This database consists of the facial geometry and texture of 150 persons, which contains 47 expression blendshapes for each person, capable of representing most expressions of the human face. All of these face blendshapes share the same topology and thus the same number of vertices. They are assembled into a rank-three tensor data (11K vertices × 150 identities × 47 expressions), and are arranged in an obvious fashion, so that each slice with varying second factor and a fixed third factor contains face vectors with the same expression (for different identities), and each slice with a varying third factor and a fixed second factor contains face vectors with the same identity (with different expressions). Any facial expression of any person (*V*) can be approximated by the tensor as:

(1) $$V=C_{r}\times_{2}W_{id}\times_{3}W_{exp}$$

where $C_{r}$ is the reduced core tensor produced by keeping the top-left corner of the original core tensor. $W_{id}$ and $W_{exp}$ are the vectors of identity weights and expression weights.

In the feature pixels extraction phase, we used the method described in [38] to locate 65 feature pixels on each viewpoint image, including the face contour, eye corners, brow boundary, mouth boundaries, nose contours and tip. These feature pixels were formulated as a shape prior to allow us to estimate accurate 3D facial contours.

The model matching phase is the main contribution of this study. In this phase, we combined the complementary characteristics of shapes from stereo matching and the 3D morphable model to extend the traditional monocular bilinear model to the multi-view-based bilinear model. An energy function with the feature prior constraint and texture constraint that were learned from multi-view images was formulated to obtain the rigid transformation of the face mesh as well as the identity and expression weights in the bilinear model.

### 3.1. Energy Formulation

**Figure 2:**
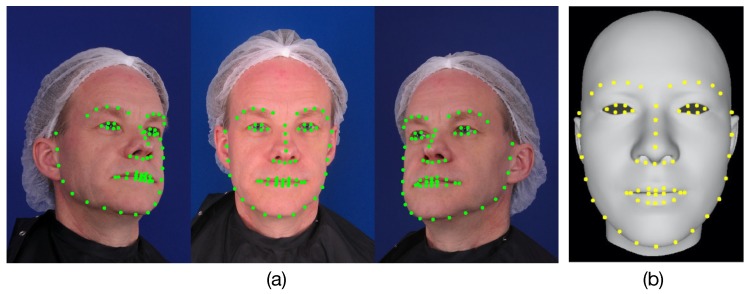
(**a**) The extracted feature pixels (green pixels) in the left, center and right viewpoints. (**b**) The initial average 3D face mesh and its corresponding landmarks (yellow points) located manually.

The estimated 3D face mesh using the multi-view based bilinear model is denoted as:

(2) $$M\left(W_{id},W_{exp}\right)=\sum_{i=1}^{m}\sum_{j=1}^{n}cr_{\left(i,j\right)}\times W_{id}\left(i\right)\times W_{exp}\left(j\right)$$

where $W_{id}$ is the *m*-dimensional vector of the identity weight and $W_{exp}$ is the *n*-dimensional vector of the expression weight. As described in [7], *m* and *n* are equal to 50 and 25 in this paper. In addition, $M^{k}\left(W_{id},W_{exp}\right)$ is the *k*-th mesh vertex in the 3D face mesh estimated according to $W_{id}$ and $W_{exp}$. As shown in Figure 2, the initialization of the bilinear model is the average 3D face mesh of all the face blendshapes (almost 3000) in the FaceWarehouse database.

To establish the connection between the 3D face mesh and the input multi-view images, we need to simulate the imaging projection principle of the cameras. Each camera is modeled as a pinhole camera and described by a rigid transformation consisting of the scaling factor $s^{i}$, the 3D rotation matrix $R^{i}$, and the translation vector $T^{i}$. The superscript i={L,C,R} indicates the left, center and right cameras. For the sake of simplicity, we assume that the camera projection is weakly perspective. Each 3D face mesh vertex $M^{k}\left(W_{id},W_{exp}\right)$ is projected to the image space as:

(3) $$P^{i}\left(M^{k}\left(W_{id},W_{exp}\right)\right)=s^{i}\times R^{i}\times M^{k}\left(W_{id},W_{exp}\right)+T^{i}$$

Based on the discussion above, in order to estimate an accurate 3D face reconstruction mesh, the energy function with the multi-view-based bilinear model, which incorporates the feature prior constraint $E_{f}$, the texture constraint $E_{t}$, and the regularization constraint $E_{r}$, learned from the multi-view images and 3D facial scanning database, can be expressed as:

(4) $$E=\lambda_{f}\times E_{f}+\lambda_{t}\times E_{t}+\lambda_{r}\times E_{r}$$

where the weights $\lambda_{f}=0.4,\lambda_{t}=0.3$ and $\lambda_{r}=0.3$ define the relative importance of each constraint. The unknowns that need to be determined are the identity weight vector $W_{id}$ and the expression weight vector $W_{exp}$ of the multi-view-based bilinear model as well as the camera calibration parameters ($s^{i},R^{i},T^{i},i=\left\{L,C,R\right\}$) of each viewpoint. The energy function Equation (4) can be easily minimized using the L-BFGS-B [39] optimization method. Each constraint is discussed in detail later.

### 3.2. Feature Prior Constraint

As described in the feature pixel extraction phase, the extracted feature pixels (green pixels) in each viewpoint are shown in Figure 2a. Each viewpoint image contains the same number and the same extraction order of the feature pixels. For example, the first feature pixel in the left viewpoint image corresponds to the first feature pixels in the center and right viewpoint images. It is clear that the extracted feature pixels located on the face contours, eye corners, brow boundaries, mouth boundaries, nose contours, and nose tip cover all of the important human facial parts and can be used to describe the human facial contours. Hence, the extracted feature pixels are used as the feature prior constraint, which is treated as a face shape prior to estimating accurate 3D face mesh contours.

To calculate the prior, we first localize a set of facial feature points (defined as landmarks and marked as yellow in Figure 2b) on the initial average 3D facial mesh following the same order of the extracted feature pixels in the images. Each landmark is a mesh vertex denoted by $M^{j}(W_{id},W_{exp}$), where *j* is the order index of landmarks. Note that because the location and extraction order of feature pixels is the same for any input viewpoint image, we only need to manually localize all landmarks in the initial average face mesh once at the beginning. To estimate a new 3D facial mesh next time, we can only directly use the landmark information.

**Figure 3:**
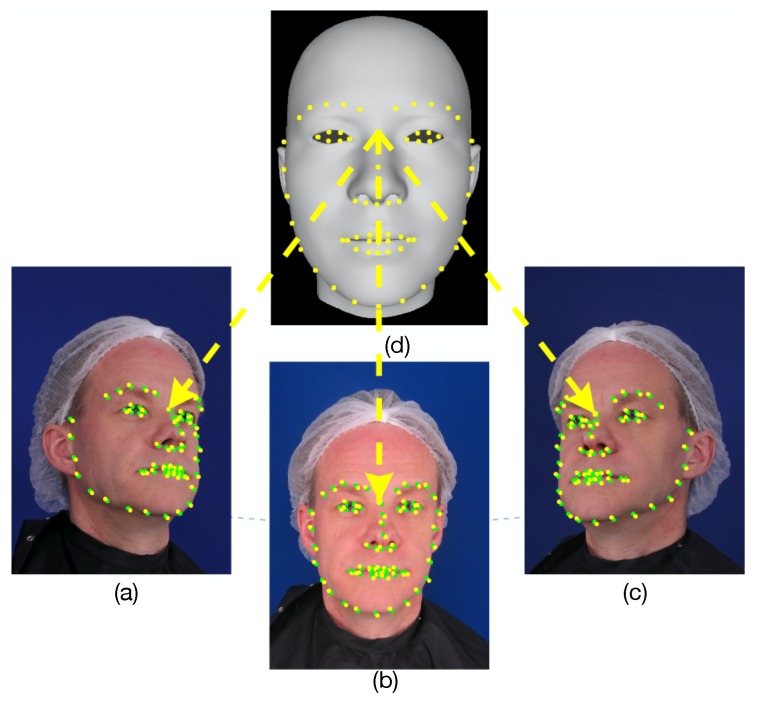
The schematic diagram of the feature prior constraint. In each viewpoint image, the projection of landmarks (yellow pixels in (**a**–**c**)) should have the same coordinate as the extracted feature pixels (green pixels).

According to the imaging projection principle of cameras, as shown in Figure 3, the *j*-th extracted feature pixels in different viewpoint images (green pixels in Figure 3a–c) can be viewed as different projections of the same *j*-th landmark (yellow pixels in Figure 3d) in the 3D facial mesh. Hence, the projection of the *j*-th landmark (yellow pixels in Figure 3a–c) should have the same coordinates as the *j*-th extracted feature pixel (green pixels in Figure 3a–c) in each image. Based on the above assumption, the feature prior constraint penalizes the distance between the projected coordinates of the landmarks and the coordinates of their corresponding extracted feature pixels. This is treated as a shape prior to obtain an accurate 3D face mesh contour and provides an estimate of the camera parameters. One feature prior constraint per viewpoint is used:

(5) $$E_{f}=\sum_{i}^{\left\{L,C,R\right\}}\sum_{j=0}^{64}\left|\right|P^{i}(M^{j}\left(W_{id},W_{exp}\right)-p_{j}^{i}{\left|\right|}_{2}$$

Here, $P^{i}$ is the projected position of the *j*-th landmark ($M^{j}\left(W_{id},W_{exp}\right)$). $p_{j}^{i}$ is the coordinate of the *j*-th extracted feature pixel. The conventional monocular bilinear model often restores a 3D face mesh using the 2D image location information of the extracted feature pixels from a single image. They do not suffer from ambiguities in texture-less or texture-repetitive regions and light illumination variation. Although the extracted feature pixels of a single viewpoint image are located at the face contours, eye corners, brow boundaries, mouth boundaries, nose contours, and nose tip, which covers all important human facial parts, the number of extracted feature pixels is limited. More seriously, the features are often affected by occlusion, which leads to inaccurate feature extraction. Hence, their results are low quality and noisy. The contours of the generated 3D face mesh may contain serious deformation because the position of extracted feature pixels is incorrect when occlusion occurs. On the contrary, we estimate the rigid transformation of the face mesh as well as the identity and expression weights in the multi-view-based bilinear model to minimize the matching error between the extracted feature pixels and the projections of the landmarks in multi-view images. This method uses the information of multi-view images to eliminate the effect of occlusion. This is possible because when some parts of the face mesh are invisible in one viewpoint, they may be visible in other viewpoints, which can provide accurate feature pixels to restore the facial contours.

### 3.3. Texture Constraint

As described above, although the feature prior constraint is viewed as a shape prior to estimating accurate 3D facial contours, the number of features is limited so that there is not enough information to estimate an accurate entire 3D face mesh (such as the cheeks) except for the face contours. In contrast, traditional shapes from stereo matching-based methods can obtain a high-quality 3D face depth map by comparing the textural difference of matching pixels between neighboring images, but they usually fail in the texture-less and texture-repetitive regions, where there is not enough visual information to obtain the correspondence.

To enhance the accuracy and robustness of the estimated 3D face mesh, an adaptive texture constraint is incorporated by fully exploring the implied 3D information between multi-view images. This assumes that the stereo-match pixels in the projected face region between multi-view images should project from the same vertex in the 3D face mesh and have a similar texture distribution. The adaptive texture constraint is the weighted sum of squared texture differences between corresponding stereo-match pixels in the multi-viewpoint images, where the correspondence is determined by the current 3D face mesh (depending on the identity weight vector $W_{id}$ and the expression weight vector $W_{exp}$ of the bilinear model as well as the camera parameters ($s^{i},R^{i},T^{i},i=\left\{L,C,R\right\}$)).

**Figure 4:**
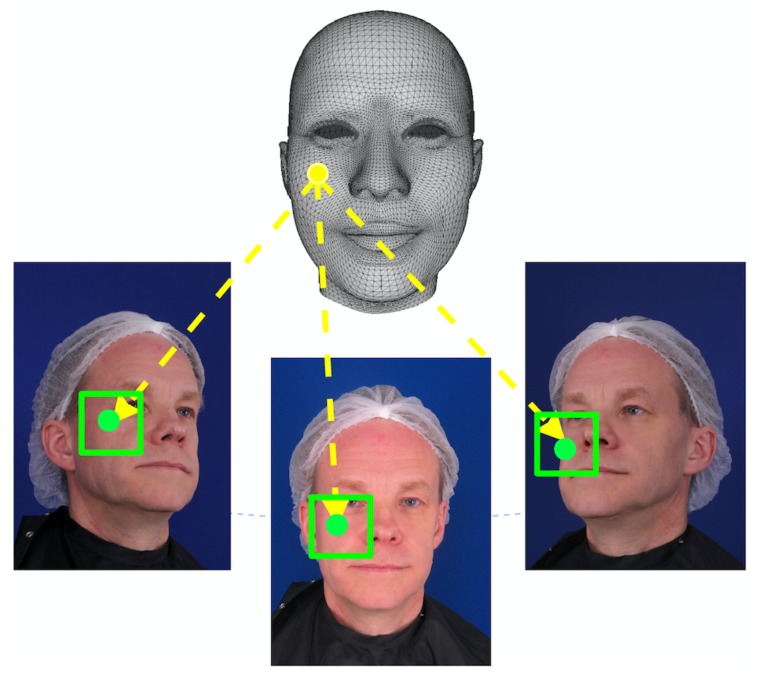
The projection of 3D face mesh vertex (yellow points) and its surrounding neighborhood patch (green pixels).

Each 3D face mesh vertex $M^{k}\left(W_{id},W_{exp}\right)$ (denoted as $V_{k}$, marked as yellow in Figure 4) is projected to each viewpoint image $P^{i}\left(V_{k}\right)$ according to the camera parameters ($s^{i},R^{i},T^{i},i=\left\{L,C,R\right\}$). Because they are projected from the same 3D mesh vertex, the projected pixels ($P^{L}\left(V_{k}\right)$, $P^{C}\left(V_{k}\right)$, $P^{R}\left(V_{k}\right)$) in the multi-view images are the corresponding stereo-match pixels. We first define a surrounding neighborhood patch $N(P^{i}\left(V_{k}\right))$ (with a radius of, for example, 5 pixels) centered at each projected pixel ($P^{i}\left(V_{k}\right)$). Let (u,v) and $\left(u^{\prime },v^{\prime }\right)$ be the image coordinates of $P^{i}\left(V_{k}\right)$ and its neighboring pixel in $N(P^{i}\left(V_{k}\right))$. The texture variance center at pixel (u,v) is calculated as:

(6) $$I^{i}\left(u,v\right)=\frac{\sum_{\left(u^{\prime },v^{\prime }\right)\in N\left(P^{i}\left(V_{k}\right)\right)}\Psi^{i}\left(u,v,u^{\prime },v^{\prime }\right)\times C^{i}\left(u^{\prime },v^{\prime }\right)}{\sum_{\left(u^{\prime },v^{\prime }\right)\in N\left(P^{i}\left(V_{k}\right)\right)}\Psi^{i}\left(u,v,u^{\prime },v^{\prime }\right)}$$

(7) $$\Psi^{i}\left(u,v,u^{\prime },v^{\prime }\right)=D\left(u,v,u^{\prime },v^{\prime }\right)H\left(u,v,u^{\prime },v^{\prime }\right)$$

where $C^{i}\left(u^{\prime },v^{\prime }\right)$ is the color value in the RGB channel, and $\Psi^{i}\left(u,v,u^{\prime },v^{\prime }\right)$ is the bilateral weight. $D(u,v,u^{\prime },v^{\prime })$ and $H(u,v,u^{\prime },v^{\prime })$ are the distance and color weights, respectively, which are defined as:

(8) $$D\left(u,v,u^{\prime },v^{\prime }\right)=\exp\left(-\frac{{\left(u-u^{\prime }\right)}^{2}-{\left(v-v^{\prime }\right)}^{2}}{2\sigma_{D}^{2}}\right)$$

(9) $$H\left(u,v,u^{\prime },v^{\prime }\right)=\exp\left(-\frac{\left|\right|C^{i}\left(u,v\right)-C^{i}\left(u^{\prime },v^{\prime }\right){\left|\right|}_{2}}{2\sigma_{H}^{2}}\right)$$

where $\sigma_{D}$ and $\sigma_{H}$ are the standard deviation of the image coordinate and the image texture space. The texture constraint is then applied to penalize the dissimilarity of the corresponding stereo-match pixel pairs, whose texture variance should be similar in all image pairs. This is calculated using the second-order texture variance term as follows:

(10) $$\begin{array}{cc} E_{t} & =\sum_{V_{k}}\left(|\right|I^{C}\left(P^{C}\left(V_{k}\right)\right)-I^{R}\left(P^{R}\left(V_{k}\right)\right){\left|\right|}_{2})\\ & -2\times \sum_{V_{k}}\left(|\right|I^{L}\left(P^{L}\left(V_{k}\right)\right)-I^{R}\left(P^{R}\left(V_{k}\right)\right){\left|\right|}_{2})\\ & +\sum_{V_{k}}\left(|\right|I^{C}\left(P^{C}\left(V_{k}\right)\right)-I^{L}\left(P^{L}\left(V_{k}\right)\right){\left|\right|}_{2}) \end{array}$$

### 3.4. Regularization Constraint

The regularization constraint is applied to prevent over-fitting of the identity weight and the expression weight. The FaceWarehouse [7] database contains more than 3000 human face blendingshapes. Because each blending shape can be approximated by the bilinear model as described in Equation (2), so we can obtain the identity weight ($W_{id}^{m}$) and the expression weight ($W_{exp}^{m}$) for the *m*-th blending shape as:

(11) $$\left\{\begin{array}{c} M_{m}^{1}\left(W_{id}^{m},W_{exp}^{m}\right)=V_{m}^{1}\\ M_{m}^{2}\left(W_{id}^{m},W_{exp}^{m}\right)=V_{m}^{2}\\ ...........\\ M_{m}^{N}\left(W_{id}^{m},W_{exp}^{m}\right)=V_{m}^{N} \end{array}\right.$$

where $V_{m}^{k}$ is the *k*-th 3D face mesh vertex from the *m*-th scanning mesh blendingshape of the face database. $M_{m}^{k}\left(W_{id}^{m},W_{exp}^{m}\right)$ is the *k*-th mesh vertex estimated from the bilinear model of the *m*-th mesh blendingshape using its corresponding identity weight ($W_{id}^{m}$) and expression weight ($W_{exp}^{m}$). When obtaining all identity weights and expression weights of entire blendshapes, we compute the mean vector and standard deviation vector of the identity and expression weights ($\mu_{id}$, $\mu_{exp}$, $\sigma_{id}$, $\sigma_{exp}$) that are denoted as the regularization term. We restrict the estimated weight vectors ( $W_{id}$, $W_{exp}$) of the multi-view-based bilinear model to be distributed around the regularization term as:

(12) $$\begin{array}{cc} E_{r}= & \sum_{i=1}^{50}{\left(\frac{W_{id}\left(i\right)-\mu_{id}\left(i\right)}{\sigma_{id}\left(i\right)}\right)}^{2}\\ & +\sum_{j=1}^{25}{\left(\frac{W_{exp}\left(j\right)-\mu_{exp}\left(j\right)}{\sigma_{exp}\left(j\right)}\right)}^{2} \end{array}$$

### 3.5. GPU-Based Optimization

The unknowns that need to be determined in the energy function Equation (4) are the identity weight vector $W_{id}$ and the expression weight vector $W_{exp}$ of the multi-view-based bilinear model as well as the camera calibration parameters ($P^{i}=\left\{s^{i},R^{i},T^{i},i=\left\{L,C,R\right\}\right\}$) of each viewpoint. Because the energy function is a nonlinear discontinuous function of the parameters, the direct optimization solution is not realistic and the iterative method is adopted.

First, we initialized all parameters. For the projection matrix, the camera calibration parameters are initialized as identity vectors. For face shape parameters, they are initialized to the shape parameters ($W_{id}$ and $W_{exp}$) of the average human face model that can be obtained by averaging 3000 human face blendingshapes in the database.

Secondly, nonlinear optimization is at the heart of many algorithms in engineering. Recently, due to the rise of general purpose graphics processing unit, it is promising to investigate the performance improvement of optimization methods after parallelization. So, the parallelized implementation of the Broyden-Fletcher-Goldfarb-Shanno (L-BFGS-B) on the GPU [39] is used to iteratively obtain each optimized parameters.

## 4. Results and Discussion

Here, a series of experiments were performed to verify the effectiveness and accuracy of the proposed method. The evaluation was composed of qualitative and quantitative analyses.

### 4.1. Quantitative Evaluation Using the ESRC Image Dataset

**Figure 5:**
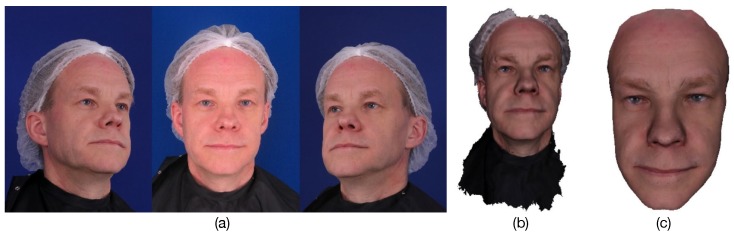
The neutral expression part of the ESRC image dataset. (**a**) From left to right is the left, center and right viewpoint images. (**b**) Raw data from depth camera. (**c**) The groundtruth of front face.

The first dataset is the neutral expression part of the ESRC image dataset [40] that contains images of 99 subjects (45 males, 54 females) from three viewpoints with ambient only lighting (see Figure 5). This should be a difficult dataset for monocular fitting, as monocular shape estimation depends on a single viewpoint image that is easily affected by having a limited number of extracted feature points and noise. Here, we performed a quantitative analysis of the proposed method using the ESRC image dataset to determine the influence of each constraint on the proposed method and these methods were compared against the monocular method [7]. Because the ESRC image dataset only provides the groundtruth of the front face (See Figure 5c), we just evaluated the metrics between the front face of our results and the groundtruth. We first reconstructed the 3D front face meshes from the ESRC image dataset and aligned them rigidly with the ground truth. Then the vertex error and the model error were used as the evaluation metrics. The vertex error whose units are millimeters (mm) is denoted as the distance between the vertices between of the reconstruction and their closest points on the groundtruth. For the model error that is denoted as the mean of all vertex errors, a lower value is better than a higher one.

**Figure 6:**
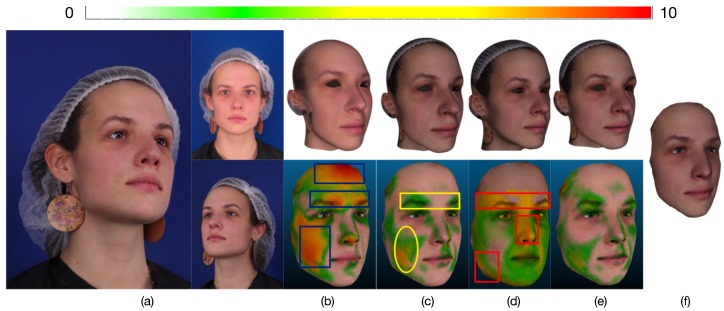
Comparison results. The vertex error (mm) is indicated by color according to the color bar. (**a**) Three viewpoint images. (**b**) Result using method [7] and its comparison result. (**c**) Result using the proposed method without texture constraint and its comparison result. (**d**) Result using the proposed method without feature prior constraint and its comparison result. (**e**) Result using the proposed method with all constraints and its comparison result. (**f**) The ground truth of front face.

One of the results of this comparison is shown in Figure 6b,e. Because the 3D face mesh was obtained from a single image, the conventional method using the monocular bilinear model [7] contains few useful face feature pixels. Moreover, its result mainly depends on the accuracy of the extracted feature pixels, which is often affected by occlusion that leads to inaccurate feature extraction. The corresponding reconstructed 3D face mesh usually contains serious deformation when the positions of the extracted feature pixels are wrong. Furthermore, it can only use the 2D image location information of face feature pixels and ignores the plentiful texture information of images, which results in low accuracy and noise (blue rectangles in Figure 6b). On the contrary, the proposed method contains the extracted feature pixels from multi-view images. It uses the information from multi-view images to eliminate the effect of occlusion. That is to say, when the location of one feature pixel is wrong or invisible in one viewpoint, its corresponding feature pixels in other viewpoints may be correct or visible. Hence, our method yields a lower error than the monocular bilinear model (See Figure 6e).

**Figure 7:**
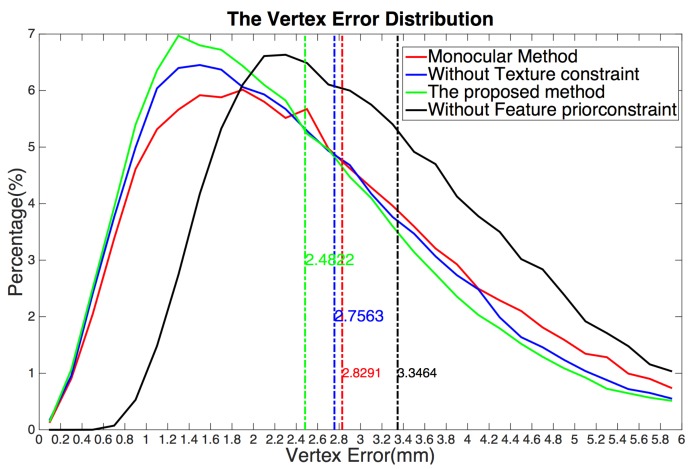
The vertex error distribution of all vertexes of ESRC dataset using different methods. The horizontal axis is the vertex error whose unit is millimeter (mm), vertical axis is the percentage of vertexes under a error threshold. For example, (4.4, 0.02) means that the percentage of vertexes with error 4.4 mm is nearly 2%. Color numbers are the model error.

Figure 7 sums up the results over all subjects in the dataset, showing that the proposed method has higher accuracy than that of the conventional method using the monocular bilinear model [7]. We can see that, on one hand, for the low vertex error region (the percentage of vertices for which the error is less than or equal to 2.4822 mm), the proposed method is the highest. A higher percentage is better. This means that our method mostly produces a low vertex error. On the other hand, for the high vertex error region (the percentage of vertices for which the error is larger than (2.4822 mm), the proposed method is also the lowest. A lower percentage indicates a better fitting. Furthermore, the model error in the ESRC image dataset is listed in Table 1. Compared with the average model error 2.8291 mm of the previous state-of-the-art monocular bilinear model [7], our method provides a model error of 2.4822 mm over all mesh vertices (nearly 400,000 vertices) for all meshes in the ESRC image datasets, which shows that our method performs almost 14% better than the conventional method with respect to precision.

Furthermore, in order to verify the precision of our method, we compared it with other state-of-the-art monocular- [41,42,43] and multi-view-based [30,37,44] approaches. According to the quantitative and qualitative evaluation results shown in Table 2 and Figure 6, it can be concluded that the proposed method is successful in the following aspects:

1. Because the feature pixels extracted from multi-viewpoints are used as the feature prior constraint, the proposed method estimates accurate contours of the generated 3D face mesh even with the effect of occlusion, where previous methods often failed. This is possible because when some parts of the face mesh are invisible in one viewpoint, they may be visible in other viewpoints, which can provide accurate feature pixels to restore the facial contours.
2. Because the adaptive texture constraint is incorporated to fully explore the implied 3D information between multi-view images, the proposed method enhances the robustness and removes the errors caused in the texture-less and texture-repetitive regions. It assumes that the stereo-match pixels in the projected face region between multi-view images should be projected from the same vertex in the 3D face mesh and have a similar texture distribution.

It is clear that the proposed method is superior to other methods with respect to the mean error rate (the accuracy increased almost 16%). Furthermore, our RMSE changes are small, which means that our method is more robust.

In addition, we evaluated the performance of each constraint of our proposed scheme. In each experiment, we omitted one part of our method and retained the remaining parts. First, we omitted the texture constraint. In this case, although the extracted feature pixels are located at the face contour, eye corners, brow boundary, mouth boundary, nose contour, and nose tip, which covers all important human facial parts, they only cover the face contours, not the entire face. Hence, the results are easily affected by noise and low quality except in the facial contour regions, which leads to reconstruction errors in the cheek region (yellow rectangles in Figure 6c) and causes the model error over the ESRC image dataset to increase sharply to 2.7563 mm, which almost decreased 11% in accuracy.

Next, we omitted the feature prior constraint, meaning that the 3D face mesh is estimated by the shape from stereo matching. Without the feature prior constraint to provide the prior knowledge of face shape contours, the results are often affected by the lighting variation. Hence, this method fails in the texture-less and texture repetitive regions where there is not enough visual information to obtain the correspondence. This leads to errors in the generated face shape contours and texture-less or texture-repetitive regions (red rectangles in Figure 6d). As listed in Table 1, the model error over the ESRC image dataset increased sharply to 3.3464 mm, which almost decreased 35% in accuracy. It is thus clear that our method significantly increases reconstruction accuracy and enhances the robustness, resulting in much better 3D face mesh when all constraints are applied. It fuses the complementary characteristics of the shape from stereo matching and the 3D morphable model to obtain better results. The feature prior constraint is used as a shape prior to allow us to estimate accurate 3D facial contours. Furthermore, the texture constraint extracts a high-precision 3D facial shape where traditional methods fail because of their limited number of feature points or the mostly texture-less and texture-repetitive nature of the input images.

**Figure 8:**
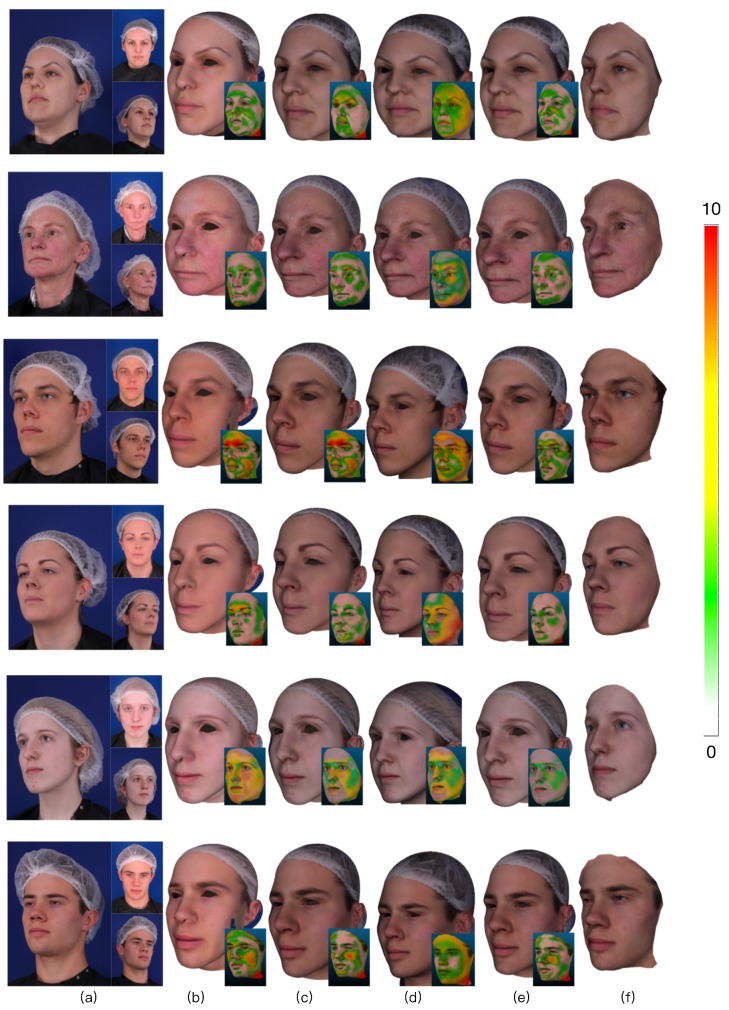
The comparison results. The range of vertex error is from 0mm to 10 mm as the color strip varies in the color bar. (**a**) Three viewpoint images. (**b**) Result using method [7] and its comparison result. (**c**) Result using the proposed method without texture constraint and its comparison result. (**d**) Result using the proposed method without feature prior constraint and its comparison result. (**e**) Result using the proposed method with all constraints and its comparison results. (**f**) The groundtruth.

Some reconstruction results are shown for a representative set of examples in Figure 8. They demonstrate that the proposed method results in a significant increase in reconstruction accuracy, resulting in a much better 3D face mesh. Meanwhile, it fully explores the implied 3D information of multi-view images, while also enhancing the robustness of the results.

### 4.2. Quantitative Evaluation Using the Morphace Image Dataset

**Figure 9:**
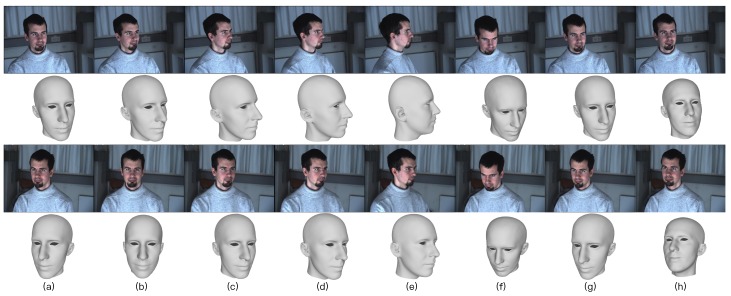
Reconstruction results under varying pose. Each column, from (**a**–**h**), is images captured simultaneously from two viewpoints (row 1 and 3) and their reconstruction meshes (row 2 and 4) from pose0 to pose7 using the proposed method.

Reconstructing the shape of a 3D face seen under varying poses should always result in the same face mesh. To investigate the robustness to varying positions and occlusions, we used the above assumption to test our method on the Morphace dataset [45], which consists of eight expressions with varying pose of 100 subjects. This dataset has the extracted feature pixel information marked in two face images of each viewpoint, so we omitted the feature extraction in the proposed method and directly used the provided feature pixel information in this experiment. We firstly estimated the 3D face mesh of each pose. Then, because there is no ground truth in this dataset, we used pose0 as the reference position and the others as the testing positions. Similarly, the estimated 3D face mesh of pose0 and the other poses were viewed as the reference mesh (Figure 9a) and testing meshes (Figure 9b–h), respectively. To quantify the robustness of our method, we computed the shape similarity between the reference mesh and each testing mesh. As is well-known, the distance between two vectors is defined as [46].

(13) $$d\left(\alpha_{1},\alpha_{2}\right)=\frac{\alpha_{1}\times \alpha_{2}}{\left|\right|\alpha_{1}{\left|\right|}_{2}\times \left|\right|\alpha_{2}{\left|\right|}_{2}}$$

The scope of the distance *d* was between −1 and 1. A higher value indicates a higher similarity. Using this definition, we defined the shape similarity ($S^{\left(r,i\right)}$) of the 3D face mesh weight vector between the reference meshes ($W_{id}^{r}$ and $W_{exp}^{r}$) and the *i*-th testing meshes ($W_{id}^{i}$ and $W_{exp}^{i}$, i=1,2…6,7) as:

(14) $$\begin{array}{c} S^{\left(r,i\right)}=0.5\times d\left(W_{id}^{r},W_{id}^{i}\right)+0.5\times d\left(W_{exp}^{r},W_{exp}^{i}\right) \end{array}$$

**Figure 10:**
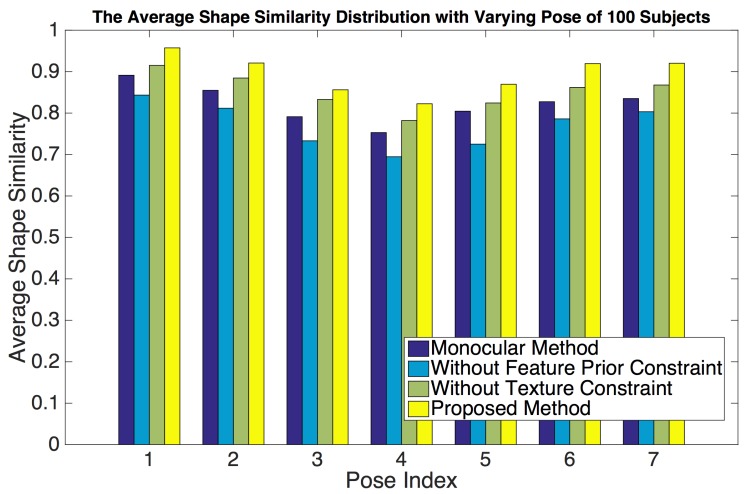
The Average Shape Similarity Distribution with Varying Pose of 100 Subjects.

The reconstruction results under each pose are shown in Figure 9 (the second and fourth rows). We compared the shape similarity between the reference mesh (pose0) and other testing meshes (pose1 to pose7) using different methods. Figure 10 clearly shows that the shape similarity is a little low in pose3, pose4, and pose5. That is because there are occlusions in these poses. So the number of available extracted feature pixels is very small. For example, the first row face image of pose4 only contains half of a human face (Figure 9e), which leads to low accuracy in feature pixel extraction. Because it hardly handles the occlusion, the accuracy of conventional monocular bilinear model-based method [7] is shapely decreased from pose3 to pose5. However, our method can obtain satisfactory result by considering the feature information from viewpoint images, which indicates the robustness against the occlusion. Furthermore, as listed in Table 3, the proposed method performs the best (highest shape similarity) in each pose and maintains a high similarity over all poses, which also indicates its robustness against varying positions of the proposed method.

### 4.3. Quantitative Evaluation with Deep Learning-Based Methods Using the ESRC and the Morphace Image Dataset

As listed in Table 4 and Table 5, we compared the performances of the proposed method and other state-of-the-art deep learning-based methods that have achieved satisfactory results. In these two tables, we can see that our results are comparable to the state-of-the-art deep learning-based algorithms. This indicates that our method is generally accurate.

### 4.4. Qualitative Evaluation with Various Expressions

**Figure 11:**
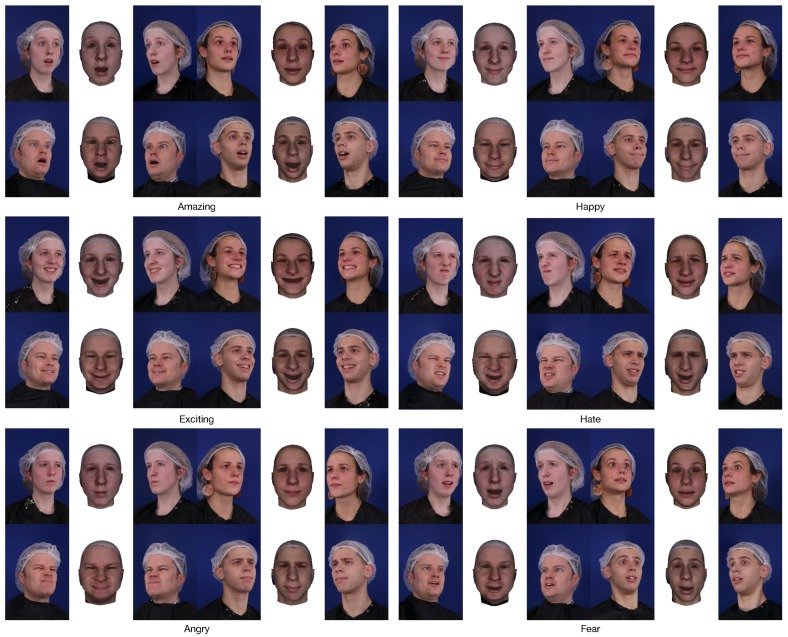
Some reconstruction results of six emotional expressions.

The third dataset is the emotional expression part of the ESRC image dataset [40] that contains images of six emotional expressions (amazement, happy, excited, hate, angry, fear) from 99 subjects (45 males, 54 females) captured from different viewpoints. Figure 11 shows some results using the proposed method. No ground truth is available for this dataset part, but we can see that our method achieves more qualitative data, which means that our method generates more realistic results that are robust against different emotional expressions.

### 4.5. Discussion

The evaluations demonstrated that the proposed method obtains robust and accurate 3D face reconstruction results. However, similar to most methods of the 3D morphable model methods, our work also used the existing FaceWarehouse [7] mesh database to build the face multi-view bilinear model. In order to reconstruct more faces with different contour features and different expressions, we need to keep enlarging the database. The proposed algorithm was implemented on a PC with Legacy $Intel^{®}$$Core^{TM}$ Processors i5-2500 (Santa Clara, CA, USA). It took approximately 0.5–1 s to obtain results for the ESRC data and Morphace datasets.

## 5. Conclusions

In this paper, we proposed a novel 3D face reconstruction method using the multi-view-based bilinear model. It incorporates the feature prior constraint and the texture constraint to explore the implied 3D information of uncalibrated multi-view images of a person. The feature prior constraint is used as a shape prior to estimate accurate 3D facial contours. Meanwhile, the texture constraint extracts high-precision 3D facial shapes where traditional methods fail. These results show a significant improvement in reconstruction accuracy, as demonstrated using ground truth data and the pose variation dataset, respectively. The evaluation results show that the proposed method can obtain satisfying results compared to the conventional methods. In the future, we intend to transform our method to a deep learning-based implementation and optimize the method so that it can be used on mobile devices.

## Figures and Tables

**Table 1:** The model error over all mesh vertices (nearly 400,000 vertices) for all meshes in the ESRC image datasets.

Method	Model Error (mm)
Monocular [7]	2.8291
Without Feature Prior Constraint	3.3464
Without Texture Constraint	2.7563
Proposed Method	2.4822

**Table 2:** Comparison of results with other state-of-the-art approaches. Model error over all mesh vertices (nearly 400,000) for all meshes in the ESRC datasets.

Method	Thies	Shi	Aissaoui	Hernandez	Piotraschke	Dai	Proposed
	et al. [41]	et al. [42]	et al. [44]	et al. [43]	et al. [30]	et al. [37]	Method
Model Error (mm)	2.7532	2.9146	2.6557	3.0015	2.3714	2.8655	2.4822
RMSE	1.7043	1.7912	1.3881	2.1514	1.8225	1.9543	1.0009

**Table 3:** The average shape similarity of 100 subjects under each pose using different methods.

	Average Shape Similarity			
	Monocular [7]	Without Feature Prior Constraint	Without Texture Constraint	Proposed Method
Pose 1	0.8913	0.8434	0.9150	0.9573
Pose 2	0.8549	0.8117	0.8846	0.9238
Pose 3	0.7912	0.7331	0.8328	0.8562
Pose 4	0.7530	0.6946	0.7820	0.8225
Pose 5	0.8046	0.7251	0.8244	0.8695
Pose 6	0.8274	0.7860	0.8620	0.9194
Pose 7	0.8351	0.8032	0.8677	0.9202

**Table 4:** Comparison results with other state-of-the-art deep learning-based approaches using the ESRC datasets. Model error over all mesh vertices (nearly 400,000) for all meshes in the ESRC datasets.

Method	Chang et al. [47]	Feng et al. [48]	Jackson et al. [49]	Tran et al. [34]	Dou et al. [50]	Proposed Method
Model Error (mm)	2.4149	2.5282	2.1375	2.1153	2.3918	2.4822

**Table 5:** Comparison results with other state-of-the-art deep learning-based approaches using the Morphace datasets. The average shape similarity of 100 subjects under each pose.

Method	Average Shape Similarity						
	Pose 1	Pose 2	Pose 3	Pose 4	Pose 5	Pose 6	Pose 7
Chang et al. [47]	0.9779	0.9221	0.9047	0.8666	0.8946	0.9114	0.9338
Feng et al. [48]	0.9634	0.9134	0.8992	0.8576	0.8839	0.9054	0.9265
Jackson et al. [49]	0.9810	0.9542	0.9317	0.8996	0.8845	0.9233	0.9503
Tran et al. [34]	0.9804	0.9487	0.9362	0.9003	0.9015	0.9277	0.9500
Proposed Method	0.9573	0.9238	0.8562	0.8225	0.8695	0.9194	0.9202
